# Technologies in Home-Based Digital Rehabilitation: Scoping Review

**DOI:** 10.2196/43615

**Published:** 2023-07-27

**Authors:** Angela Arntz, Franziska Weber, Marietta Handgraaf, Kaisa Lällä, Katariina Korniloff, Kari-Pekka Murtonen, Julija Chichaeva, Anita Kidritsch, Mario Heller, Evanthia Sakellari, Christina Athanasopoulou, Areti Lagiou, Ioanna Tzonichaki, Iosune Salinas-Bueno, Pau Martínez-Bueso, Olga Velasco-Roldán, Ralf-Joachim Schulz, Christian Grüneberg

**Affiliations:** 1 Division of Physiotherapy Department of Applied Health Sciences University of Applied Health Sciences Bochum Bochum Germany; 2 Faculty of Human Sciences University of Cologne Cologne Germany; 3 Department of Rehabilitation, Physiotherapy Science & Sports University Medical Center Utrecht Utrecht Netherlands; 4 Institute of Rehabilitation Jamk University of Applied Sciences Jyväskylä Finland; 5 Institute of Health Sciences St. Pölten University of Applied Sciences St. Pölten Austria; 6 Department of Media & Digital Technologies St. Pölten University of Applied Sciences St. Pölten Austria; 7 Department of Public and Community Health Laboratory of Hygiene and Epidemiology University of West Attica Athens Greece; 8 Department of Occupational Therapy University of West Attica Athens Greece; 9 Health Research Institute of the Balearic Islands (IdISBa) Palma de Mallorca Spain; 10 Department of Nursing and Physiotherapy University of the Balearic Islands Palma de Mallorca Spain; 11 Department of Geriatric Medicine St. Marien-Hospital Cologne Germany

**Keywords:** digital rehabilitation, digital technologies, home-based rehabilitation, digital health intervention, scoping review, artificial intelligence, AI, machine learning, COVID-19 pandemic, mobile app, remote health, mobile phone

## Abstract

**Background:**

Due to growing pressure on the health care system, a shift in rehabilitation to home settings is essential. However, efficient support for home-based rehabilitation is lacking. The COVID-19 pandemic has further exacerbated these challenges and has affected individuals and health care professionals during rehabilitation. Digital rehabilitation (DR) could support home-based rehabilitation. To develop and implement DR solutions that meet clients’ needs and ease the growing pressure on the health care system, it is necessary to provide an overview of existing, relevant, and future solutions shaping the constantly evolving market of technologies for home-based DR.

**Objective:**

In this scoping review, we aimed to identify digital technologies for home-based DR, predict new or emerging DR trends, and report on the influences of the COVID-19 pandemic on DR.

**Methods:**

The scoping review followed the framework of Arksey and O’Malley, with improvements made by Levac et al. A literature search was performed in PubMed, Embase, CINAHL, PsycINFO, and the Cochrane Library. The search spanned January 2015 to January 2022. A bibliometric analysis was performed to provide an overview of the included references, and a co-occurrence analysis identified the technologies for home-based DR. A full-text analysis of all included reviews filtered the trends for home-based DR. A gray literature search supplemented the results of the review analysis and revealed the influences of the COVID-19 pandemic on the development of DR.

**Results:**

A total of 2437 records were included in the bibliometric analysis and 95 in the full-text analysis, and 40 records were included as a result of the gray literature search. Sensors, robotic devices, gamification, virtual and augmented reality, and digital and mobile apps are already used in home-based DR; however, artificial intelligence and machine learning, exoskeletons, and digital and mobile apps represent new and emerging trends. Advantages and disadvantages were displayed for all technologies. The COVID-19 pandemic has led to an increased use of digital technologies as remote approaches but has not led to the development of new technologies.

**Conclusions:**

Multiple tools are available and implemented for home-based DR; however, some technologies face limitations in the application of home-based rehabilitation. However, artificial intelligence and machine learning could be instrumental in redesigning rehabilitation and addressing future challenges of the health care system, and the rehabilitation sector in particular. The results show the need for feasible and effective approaches to implement DR that meet clients’ needs and adhere to framework conditions, regardless of exceptional situations such as the COVID-19 pandemic.

## Introduction

### Background

Rehabilitation is an essential part of caring for people with acute or chronic health conditions, impairments, or injuries that limits functioning [1]. It is estimated that 2.5 billion people worldwide live with health conditions that benefit from rehabilitation [1]. Owing to population growth, aging, and the increasing number of people with chronic diseases and disabilities, the need for rehabilitation is steadily increasing worldwide [1].

Two of the most challenging aspects of rehabilitation are the high costs of inpatient and long-term rehabilitation programs [2] and the poor continuity of rehabilitation when patients are transferred to their homes [3,4]. To address these challenges, a shift in rehabilitation from inpatient care or rehabilitation centers to home settings is essential [5,6]. Therefore, various models have been developed to offer early home-based rehabilitation [7,8]. However, for effective home-based rehabilitation, sufficient support must be provided to clients (persons who receive health care services) and health care professionals (persons who provide health care services).

Incorporating new digital technologies into rehabilitation could help to meet these demands. Digital rehabilitation (DR) can be defined as using digital technologies as a part of the rehabilitation process [9]. DR aims to optimize functioning and reduce disability of individuals with health conditions in interaction with their environment [9]. This includes, but is not limited to, the use of tele- and remote rehabilitation applications and services, automatic services, robot-assisted technologies, wearables, emails, video, speech, and SMS text messaging solutions [9]. By using tele- and remote rehabilitation applications and services, for example, sensors and wearables, opportunities exist for monitoring clients’ health status at home [10-12]. In addition, DR improves rehabilitation outcomes in clients with heart failure, diabetes, and respiratory disease [13]. They also help clients to manage pain; increase their physical activity; and improve mental health, diet quality, and nutrition [13]. Furthermore, it appears that some parts of the DR are cost-effective [13]. With the combination of commercially available technologies, Internet of Things (IoT), and artificial intelligence (AI), there is also the possibility of remote health assessments and personalized rehabilitation interventions [14]. In addition, DR can have a positive impact on self-management [15-17]. Self-management aims to improve clients’ ability to manage their disability and improve their lifestyles. It underlines the active participation of clients, emphasizing the interactive and collaborative relationship between clients and health care professionals. Similarly, an important aspect of self-management is client responsibility, which is particularly important in home-based rehabilitation [18].

However, for the effective application of DR, it is not only important to identify digital technologies and their potential applications that can be integrated into home-based rehabilitation but also essential to equip health care professionals with the skills needed to provide high-quality rehabilitation, conferring them with the potential to develop the field multi-professionally.

The COVID-19 pandemic has exacerbated the challenges faced by individuals in need of rehabilitation [19]. In addition, there has been increased pressure on higher education institutions and health care professionals to develop DR practices that meet the needs of target populations [20].

To develop and implement DR solutions that meet client needs and ease the growing pressure on the health care system, it is necessary to provide a broad overview of existing, relevant, and future solutions shaping the constantly evolving market of technologies for home-based DR.

### Objective

The scoping review aimed to identify digital technologies for home-based rehabilitation, predict new and emerging trends in DR, and report the influences of the COVID-19 pandemic on DR.

## Methods

### Overview

The scoping review was performed based on an adapted framework described by Arksey and O’Malley [21] by adding the improvements proposed by Levac et al [22]. This framework includes 5 phases: identifying research questions, determining relevant studies, selecting studies, charting data, and consulting with key stakeholders and experts. The scoping review was reported consistent to the PRISMA-ScR (Preferred Reporting Items for Systematic Reviews and Meta-Analyses extension for Scoping Reviews; Multimedia Appendix 1). A protocol of this scoping review does not exist.

### Identification of Research Questions

As the first step, the following research questions were identified:

- Research question 1: What are the types of existing new or emerging digital technologies in home-based rehabilitation?
- Research question 2: Which trends can be identified for the home-based DR technologies and what are their advantages and disadvantages?
- Research question 3: How does the COVID-19 pandemic influence the development of DR?

### Identification of Relevant Studies

To identify references related to the research questions, a scientific database search in PubMed, Embase, CINAHL, PsycINFO, and the Cochrane Library was performed in July 2021.

The search strategy was first developed for PubMed and adapted to each database by using keywords; their synonyms; and related terms of “rehabilitation,” “home-based,” and “digital technologies.” These terms were connected with the Boolean operators “AND” and “OR” to obtain a wide spectrum of results from the various databases. The full search strategy for each database is presented in Multimedia Appendix 2. The search for relevant references was designed in 2 phases. The first phase involved searching references published from January 2015 to July 21, 2021. In the second phase, an additional search was performed in January 2022 to retrieve recent publications.

To complement the findings regarding the trends of home-based DR, as well as the impact of the pandemic, gray literature sources were searched for unpublished materials in the native languages by the member institutes of the project “Competences for the new era of user-driven Digital Rehabilitation (DIRENE)” (Greek, Spanish, Finnish, German, and English). The gray literature was searched using Google, Google Scholar, Yahoo, OpenGrey, specific websites from each country’s government, thesis repositories, and university library websites that offer a comprehensive list of gray literature databases.

### Study Selection

Study selection was performed using the web-based software, Covidence (version 2021; Veritas Health Innovation) [23]. After a training period, 7 independent researchers from the DIRENE consortium performed study selection based on the eligibility criteria presented in Textbox 1. For every reference, 2 randomized researchers individually assessed the reference in terms of inclusion or exclusion. In cases of disagreement, a third researcher was included to resolve conflicts.

**Eligibility criteria for study selection.**

**Inclusion**

Article type

- Every article type that is existing as a full text

Language

- English

Publishing date

- First phase: references published since January 1, 2015
- Second phase: references published since July 21, 2021

Technology

- Digital technology directly related to home-based rehabilitation
- Fully developed devices, applications, software, or prototypes and ideas

Intervention

- Every intervention in which a digital technology was used

**Exclusion**

Article type

- Articles that were not available as full texts via common publishers, universities databases or directly requested from the publishing authors

Language

- Other languages than English

Publishing date

- First phase: references published before January 1, 2015
- Second phase: references published before July 21, 2021

Technology

- Nondigital technologies
- Technologies used after invasive procedures (eg, surgery to implant a device)

Intervention

- Intervention via telephone as a stand-alone intervention
- Intervention in a laboratory setting

### Data Extraction and Data Charting

A bibliometric analysis was performed as the first overview of the included publications. The software Zotero (version 6.0.10; Roy Rosenzweig Center for History and New Media, George Mason University) [24] was used to indicate the number of publications per year and the journals of the included references.

To identify new and emerging technologies for home-based rehabilitation, a co-occurrence analysis was performed. The software VOSviewer (version 1.6.18; Leiden University) [25] was used for the analysis. The co-occurrence analysis was intended to map the results of the included publications, categorize the technologies mentioned in the publications, and provide a visualization of the output [26]. For this purpose, a Research Information Systems (RIS) file of all included publications was uploaded to the program, and a text-based analysis was performed using a binary counting method of the most frequently occurring keywords [26]. On the basis of the relevance score, the program automatically selected the most applicable keywords for the final analysis.

Furthermore, a full-text analysis of all included reviews was conducted to filter the trends for home-based DR technologies and to present its advantages and disadvantages. The analysis aimed to extract relevant information about the technologies in terms of the goal, specification and application, population, and advantages and disadvantages of the technologies.

The data of the gray literature search were analyzed to complement the results of the review analysis and to determine the influence of the COVID-19 pandemic on the development of DR. Each participating institution used an extraction sheet to obtain the information with respect to the research questions. All data were then summarized according to the following categories: type, aim, description of the technology, target population, and statements regarding the influence of the COVID-19 pandemic.

### Stakeholder Consultation

The aim of the stakeholder consultation was to complement the results regarding the trends of digital technologies and the impact of the pandemic on DR from different perspectives. Therefore, a meeting with key stakeholders and experts was organized by 4 of the 5 collaborating universities of the DIRENE consortium (Greece, Finland, Austria, and Germany). Participants included clients with experience or specific interest in using digital technologies at home (10/56, 18%); rehabilitation professionals (13/56, 23%); experts from companies for future trends in health care (9/56, 16%); experts in digitalization (11/56, 20%); and representatives of public health administrations (6/56, 11%), social and welfare departments (4/56, 7%), and national platforms for digitalization in rehabilitation (3/56, 5%).

The meetings consisted of a short introduction to the project and the presentation of the preliminary results of the review. Stakeholders shared their views on the identified trends in digital technologies used in rehabilitation. They discussed the potential synthesis of technologies in home-based rehabilitation and examined factors influencing DR. All the information was summarized in a final standardized report by each participating institution. The results were then analyzed in line with the Framework Approach [27]. On the basis of the research questions, a deductive approach was used to form the key themes and subthemes. After reviewing the results of the stakeholder consultation for each participating institution, the subthemes were adjusted inductively. Subsequently, the results of each stakeholder consultation were coded. Each code in a report was then summarized, abstracted, and tabulated for each subtheme. Then, the statements of all stakeholder consultations were summarized per key theme.

## Results

**Figure 1:**
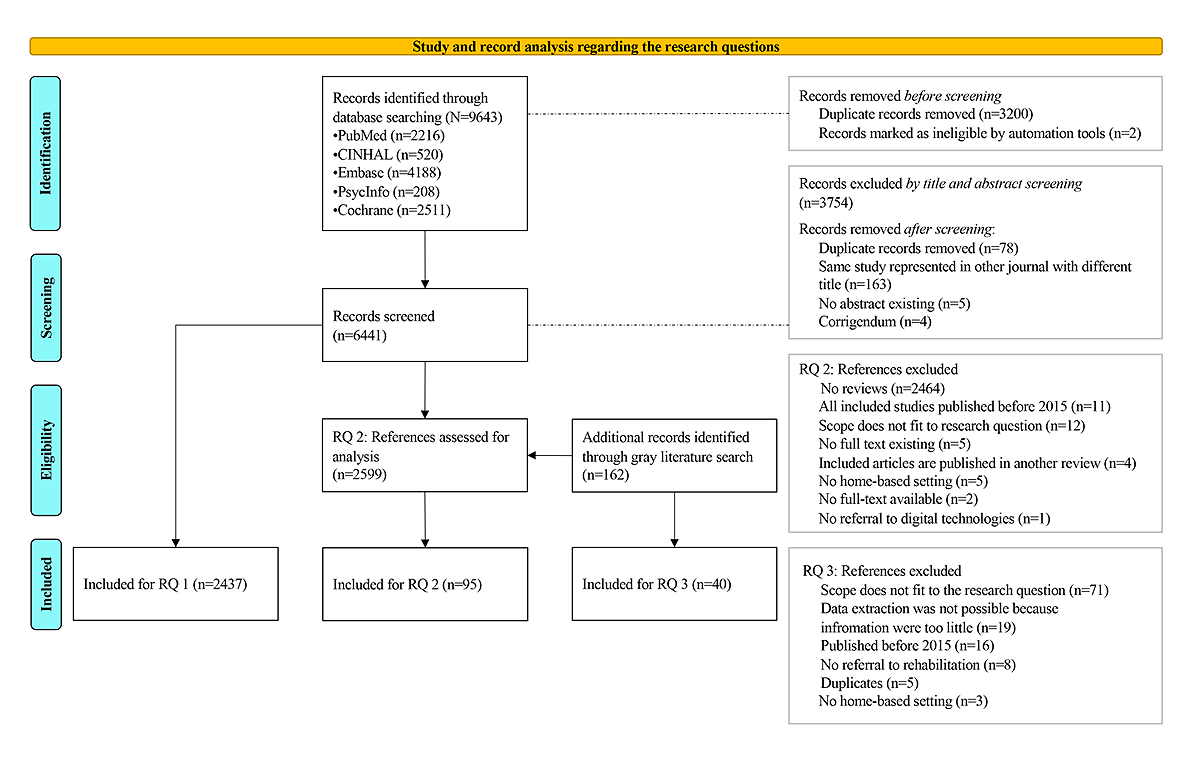
Flowchart of the paper-selection process. RQ: research question.

The process of paper selection for each research question is shown in Figure 1.

### Research Question 1: New or Emerging Digital Technologies in Home-Based Rehabilitation

Bibliometric and co-occurrence analyses were performed to provide an overview of the included references and identify new or emerging technologies in home-based rehabilitation. For these analyses, 2437 records were included.

All 2437 included records for the bibliometric and co-occurrence analyses were published between January 2015 and January 2022. Over the past years, the number of publications has steadily increased annually, from 211 (8.66%) in 2015 to 544 (22.32%) in 2021. In addition, most research papers were published in 2021 (n=544, 22.32%).

**Figure 2:**
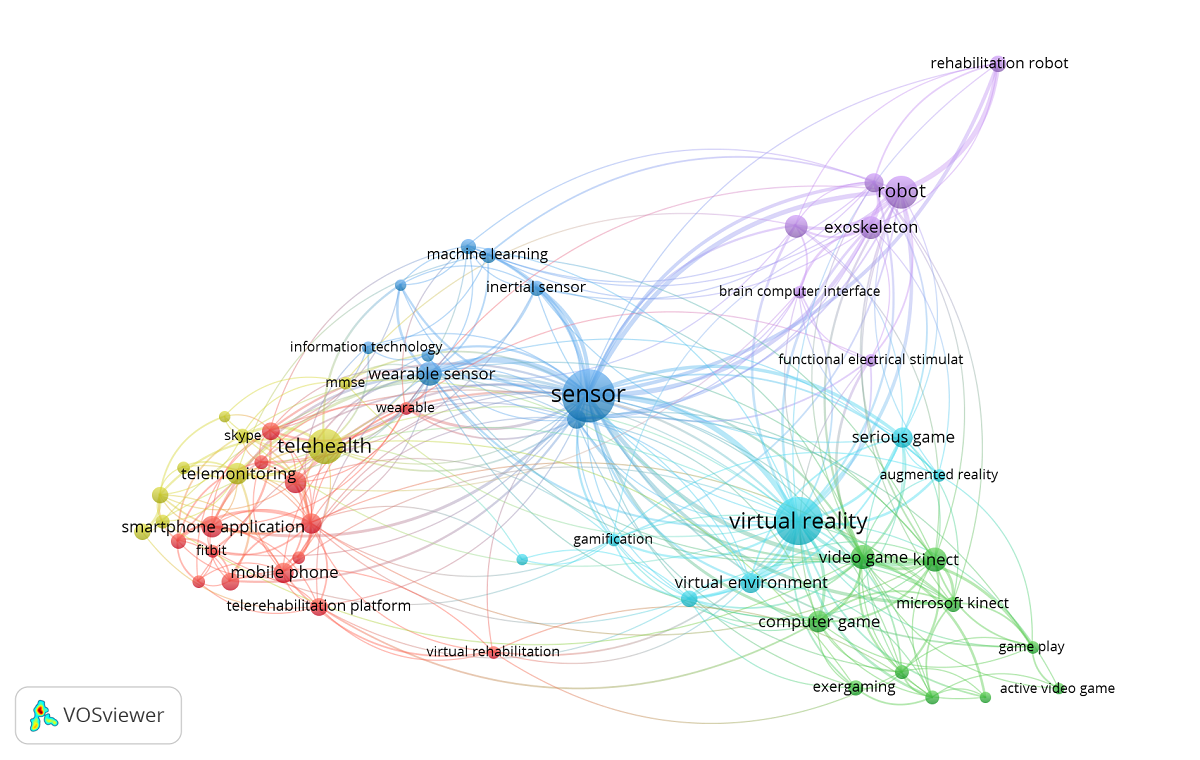
VOSviewer co-occurrence analysis of the most mentioned keywords referring to home-based rehabilitation. Map generated in VOSviewer version 1.6.18; Leiden University.

In total, 53 keywords with a frequency of ≥10 were selected from 59,718 keywords, and a co-occurrence analysis was performed, as shown in Figure 2.

As presented in Figure 2, the research themes of home-based DR can be divided into 6 clusters: sensors (blue), robotics (purple), gamification (green), virtual reality (VR) and augmented reality (AR; turquoise), mobile apps (red), and digital platforms (yellow). Each node represents a keyword. The size of the node indicates the number of occurrences of that keyword, and the link connecting the 2 nodes indicates that a keyword appears in common with another keyword. The thickness of the connection line indicates the strength of co-occurrence between the 2 keywords. The visualization indicates that the keyword “sensor” is the most frequently occurring term throughout the included publications compared with other keywords represented in this cluster.

Further analysis of keywords that occurred ≥10 times provided an overview of the mentioned populations in which the identified technologies were used in home-based rehabilitation, as shown in Table 1.

It can be seen that the cumulative sum of stroke and related synonyms are the most occurring conditions related to home-based rehabilitation among the publications. Chronic obstructive pulmonary disease (COPD) and its synonyms ranked second. The third most common disease mentioned in the included publications is multiple sclerosis (MS).

**Table 1:** Population or symptoms groups sorted per occurrence in the bibliometric analysis.

Population	Occurrences, n
Stroke patient	100
Chronic stroke	38
Chronic stroke patient	29
Chronic stroke survivor	21
Poststroke patient	15
COPD^a^	102
Chronic obstructive pulmonary disease	77
Severe COPD	13
MS^b^	116
MS patient	17
Parkinson	98
Parkinson disease	13
Spinal cord injury	61
Traumatic brain injury	48
Aphasia^c^	30
Hemiplegia^c^	16
Cardiovascular disease	55
Heart failure	55
Myocardial infarction	26
Coronary heart disease	23
Hypertension^c^	22
Acute coronary syndrome	19
Chronic heart failure	18
Ischemic heart disease	17
Coronavirus disease	57
Chronic respiratory disease	12
Dyspnoea^c^	14
Knee osteoarthritis	30
Osteoarthritis	25
Hip fracture	21
Chronic low back pain	17
Knee injury^c^	13
Total hip arthroplasty	13
Total knee arthroplasty	12
Depressive symptom^c^	32
Breast cancer	16
Diabetes	16
Alzheimer	17

^a^COPD: chronic obstructive pulmonary disease.

^b^MS: multiple sclerosis.

^c^The given groups represent symptoms that occurred in the bibliometric analysis.

### Research Question 2: Trends in Home-Based DR and its Advantages and Disadvantages

#### Overview

To identify the trends for home-based DR technologies and display their advantages and disadvantages, 95 reviews (systematic reviews: n=51, 54%; reviews: n=44, 46%) were included (Figure 1) in the review analysis. For the technologies “brain-computer interface” and “machine learning,” no reviews could be found. However, the co-occurrence analysis showed results for these 2 technologies (Figure 2). All references were screened again these topics. Therefore, 22 articles were additionally considered for the analysis.

It is essential to note that each publication addressed one or more types of technologies that are often used in combination with another. For example, exergames or serious games that are used with a head-mounted display are grouped in the category “virtual/augmented reality,” and the games in which a client plays in a virtual environment without using technology for an immersive experience are categorized as “gamification.” Likewise, the technology was not named in either category to avoid duplication.

To provide a definition for each identified technology, the DIRENE consortium developed definitions based on the current literature using a Delphi process until a consensus was reached [28] (Multimedia Appendix 3 [1,29-46]).

Table 2 provides an overview of the identified technologies for home-based DR and shows their advantages and disadvantages.

**Table 2:** Summary of the identified technologies discussed in home-based rehabilitation ordered by the specification of technology, specification of application, advantages, and disadvantages or limitations.

Category of technologies and specification of technologies		Specification of application		Advantage	Disadvantage or limitation		Included reviews or articles
**Sensors**							
	Inertial sensors	Measuring, assessing, capturing, and tracking movement, motor activity, gait analysis, falls, blood flow, and respiratory rate; movement coding for control keyboards and displays; control and implementation of tasks and human-machine interfaces such as wheelchairs, smart shoes, and robots; and gesture recognition to aid communication between people with hearing impairments and listeners	Relatively inexpensive, portable, and user-friendly; provide sufficiently accurate and fast movement data for rehabilitation analysis and evaluation; simple principles of operation		Loss of accuracy due to factors such as position of sensor placement, reliability of skin attachment, or an interaction effect with the sensors	[47-49]	
	IMUs^a^	Measuring, assessing, capturing, or tracking movement and posture; predicting falls; and providing biofeedback	Small design, low cost, simple handling, capable of delivering accurate and valid analysis, and captures 3-dimensional linear accelerations from accelerometers and angular velocities from gyroscope. Combination of linear and rotational data enables a more complete picture of motion, as it has many *df*		Lack of validation in terms of capturing posture and motion and providing biofeedback. To measure accurately, at least 3 sensors are necessary. Multiple sensors require connection via wires and attachment to the body, often by strapping, and they may be challenging to remove and reattach	[47,50- 54]	
	Accelerometer	Measuring, assessing, capturing, and monitoring movement, motor activity, physical activity, posture, respiratory rate, steps, falls, sleep, and gait analysis and providing biofeedback	Capture linear acceleration data in 1-3 planes of motion, can be used when magnetic interference is a concern, uses the gravity vector as a reference, can be easily attached to clients at low cost, and simple principles of operation		Sparse data-collection, often multiple sensors required	[48,49, 51,53, 55-57]	
	Gyroscope	Measuring, assessing, and capturing movement and motor activity	Can be easily attached to clients		Have no reference as gravity and are therefore unable to establish an initial state, leading to error accumulation	[53,56, 57]	
	Infrared sensors	Measuring, assessing, capturing, and monitoring motor activity and posture	User-friendly		No data could be extracted from the included literature	[47,58]	
	Flex sensors	Control and implementation of tasks and human-machine interfaces such as wheelchairs, smart shoes, and robots; and gesture recognition to aid communication between deaf people and listeners	No data extracted from included literature		No data extracted from included literature	[47,51]	
	GPS and smartwatch	Measuring and monitoring steps and physical activity	No data extracted from included literature		No data extracted from included literature	[55,59]	
	Photo sensors	Measuring temperature, respiratory rate, and emotion recognition	User-friendly		No data extracted from included literature	[56,58]	
	(Vision) cameras	Measuring, assessing, and capturing movement, heart and respiratory rate, gait analysis, and blood flow; control and implementation of tasks and human-machine interfaces such as wheelchairs, smart shoes, or robots, and emotion recognition	Available commercially and at low costs		When measuring motion, vision cameras without an optical motion–tracking system provide 2D information about the captured scene; lack of the third dimension’s information imposes limits on the evaluation accuracy	[47,48, 56]	
	EMG^b^	Measuring heart rate, gait analysis, movement coding for control keyboards and displays; gesture recognition to aid communication between deaf people and listeners; and recognition of fascial expressions	Available commercially and at low costs		To measure bioelectric signals, often inertial sensors have to be added	[47,50]	
	Microphone	Measuring and tracking social activity	Available commercially and at low costs		No data extracted from included literature	[47,56]	
	Electrodes	Prevention of dementia, treatment for behavioral change, activating muscle contraction, and biofeedback	No data extracted from included literature		Multiple sensors are often required	[47,60, 61]	
	Chemical and glucose sensors	Glucose monitoring	No data extracted from included literature		Requires a regular calibration to reduce errors	[47]	
**Robotics and brain-computer interface**							
	Robotic gloves	Measuring, assessing, capturing, and tracking motor function; supporting hands and finger movement; strengthening muscular activity and hand and finger coordination; and assisting ADLs^c^	Promotes engagement and motivation in therapy		Different operating characteristics, high costs, and predominantly passive in nature when used without a therapist	[62-64]	
	Exoskeleton for upper and lower limbs	Active, passive, and triggered assistance of movement; implementing movement; and gait training	Promotes engagement and motivation in therapy, delivers high-intensity training compared with therapist-only training and assists or helps to perform movement even if the client cannot initiate movement		General: individual physical characteristics (cognitive, communication, visual problems, and motor impairments) may limit the use of exoskeletons; the need to be assisted by others to operate the rehabilitation robot at home; skills required to operate the system; high purchase and maintenance costs; and limited accessibility. Exoskeletons lower limbs: high risk of falls, there is a need to learn how to use the exoskeleton while walking, and there are special adaptation requirements when using the device	[54,63- 67]	
	Robotic device for upper and lower limbs	Active and passive assistance, supporting movement, improving movement, and assisting gait	Able to generate a wide variety of forces and motions and deliver measurable doses and intensities of therapy		Require large amounts of physical space and appropriate facilities such as tables and chairs for setup; some robots generate large forces, which can create theoretical safety concerns during unsupervised use at home	[49,54, 62,64- 66]	
	Brain-computer interface	Active and passive assistance of movement, implementing movement, supporting ADLs, and enable or support communication with environment	Home use is possible and enables movement through brain activity		In its infancy, low usability rate, and often costly	[68-76]	
	Social robots	Supporting ADLs	Could reduce loneliness, older individuals are willing to use robotic technologies		Acceptance rate in healthy participants is low	[77]	
**Gamification**							
	Serious games	Improving balance; gait; mobility; postural control; motor, physical, and cognitive functioning; adherence; and self-management	Promote engagement and motivation in therapy; specific exercises are provided based on clients’ aims. The training process is monitored, and the training plan is adapted accordingly		Additional hardware is costly, requires certain skills for client and the therapist to operate, health professional should monitor the compliance with the prescribed tasks at a regular basis to make adaptions to the rehabilitation plan, and diverse acceptance rate	[54,78- 80]	
	Exergames	Improving balance; gait; mobility; postural control; and motor, physical, and cognitive function; preventing falls; and reducing symptoms of chronic respiratory diseases	Commercially available, promote engagement and motivation in therapy, and many are low-cost systems		Specific guidance and tailored interventions to the clients’ needs is often lacking. Devices are not always designed for people with disabilities. Diverse adherence rate	[52,54, 59,81- 92]	
**Virtual and augmented reality**							
	Augmented reality	Improving physical functioning, range of motion, and gait	Promotes engagement and motivation in therapy, no proof to cause symptoms of “simulator sickness”		In its infancy and requires further investigation with regard to their effectiveness. Dizziness may occur during use	[93,94]	
	Virtual reality	Improving physical functioning, fitness, balance, postural control, vestibular dysfunction, and anxiety	Promotes engagement and motivation in therapy. Some systems are commercially available at low costs		Potential side effect known as “motion sickness” may occur during use. Fully immersive systems are not commercially available and not at low costs	[93,95- 99]	
**Digital and mobile apps**							
	App	Measuring, assessing, capturing, and tracking rehabilitation process and health behavior, medication and rehabilitation adherence, and active and passive movement; providing and performing assessments; promoting self-management, physical activity, and healthy lifestyle; reducing falls; improving physical functioning, trunk control, dexterity, cognitive and language skills, and mobility; providing psychosocial support, coaching, secondary prevention; and obtaining support from other people	Low cost, commercially available, provides access to some rehabilitation measure, beneficial to combine app solutions (eg, for diagnosis, intervention, or monitoring), increase engagement in therapy, and supports connection between health care professional and client through real-time transmission of health data		Accuracy of measuring ROM^d^ is not tested or validated yet. Access and use can be different between countries due to cultural background, availability of high-speed connection, and trust in health care professionals. Correct use of digital technology may be affected by health condition itself (eg, motor disability, visual impairment, psychiatric comorbidities, cognitive dysfunction). Some of the apps could only be used in combination with another technology (eg, smartwatch). Some apps did not offer a platform to facilitate interaction with health professionals; some apps are outdated; some apps lack of disclosing sponsorship, authors’ affiliations, credentials, and sources or references of information; and some apps do not always cover all the rehabilitation needs for the client. Security aspects are not always considered	[49,52, 55,59, 100-114]	
	Web-based program	Measuring, assessing, capturing, and tracking, rehabilitation process, health behavior, and medication and rehabilitation adherence; providing and performing assessments; promoting self-management, physical activity, and healthy lifestyle; improving physical functioning, balance, postural control, endurance, strength, and cognitive skills; obtaining support from other people	Real-time feedback is possible, low cost, commercially available, access to some rehabilitation measures, beneficial to combine app solutions (eg, for diagnosis, intervention, or monitoring), engagement in therapy, supports connection between health care professional and client through real-time transmission of health data		Access and use can be different between countries due to cultural background, availability of high-speed connection, and trust in health care professionals; correct use of digital technology may be affected by health condition itself (eg, motor disability, visual impairment, psychiatric comorbidities, and cognitive dysfunction); sometimes unreliable connections	[49,52, 55,59, 100,104, 107-111, 113,115- 122]	
	Videoconference	Promoting self-management and healthy lifestyle, improving mobility and physical functioning, monitoring rehabilitation process and exercises, and obtaining support from other people	Remote rehabilitation and real-time feedback possible, supports motivation in rehabilitation, low cost, commercially available, possibility to reduce duration of inpatient hospitalization, may reduce costs		Workability is not always practical, lack of physical interaction between clients and therapists, technical skills are necessary for the use of some services. Main policy challenges related to home-based telerehabilitation are not yet fully resolved (eg, costs, reimbursement, data protection, liability, and system security). Access and use can be different between countries due to cultural background, availability of high-speed connection, and trust in health care professionals. Correct use of digital technology may be affected by health condition itself (eg, motor disability, visual impairment, psychiatric comorbidities, and cognitive dysfunction)	[49,52, 54,59, 61,103, 104,113]	
**Internet of Things**							
	Smart homes and ambient-assisted living	Detecting and preventing mild cognitive impairments and dementia, improving cognitive functioning, supporting tasks of daily living, and monitoring health status	Monitoring clients’ health status in their natural environment		Use of the technology is complex for clients with disabilities. Technology could be expensive—costs for installation, repair and maintenance occur, ethical considerations. Privacy concerns and clients’ safety are often discussed issues	[47,58, 101,115]	
	Living labs	Assessing motor performance	Testing is possible in the home environment, records sufficient objective measures, and variable test administration by clients is bypassed		Clients may not be fully informed about the possibilities and limitations of the technologies or are not cognitively able to understand their implications; too many data are collected, most important information has to be filtered, may violate client privacy, and challenging to comply with ethical and data protection guidelines. Sometimes additional technologies have to be attached to clients such as electrodes; this can be demanding and uncomfortable (eg, constant use and attachment or detachment of electrodes)	[115,123]	
**AI^d^ and machine learning**							
	AI and machine learning	Providing individualized therapy, trainings plan, motion feedback in real time, and classification of movement	Have the potential to predict adherence and client conditions		In its infancy; some decisions could be taken over from AI and machine learning algorithms; and acceptable model accuracy can be reached after using the technology multiple times	[124-136]	
	Chatbots and conversational agents	Organizing rehabilitation process, treatment of mental disorders, and assisted living	Rehabilitation management is supported, entertaining tool for rehabilitation, low cost, and no stigmatization in mental disorder treatment		In its infancy, more research is needed; most conversational agents are not commercially available; some individuals may become overly attached, some chatbots give inappropriate responses related to the health problem; there are no laws and regulations for the use of chatbots, and the legal responsibility for adverse events related to chatbots has not yet been clarified	[121,137- 139]	
	Virtual humans	Secondary prevention, promoting physical activity, and treatment of mental illness	Appear natural for human-machine interaction and give the illusion of liveliness of interaction with a real person, exude trustworthiness and credibility		There is the fear of replacing a health care professional, which can lead to considerable disadvantages	[121]	

^a^IMU: inertial measurement unit.

^b^EMG: electromyography.

^c^ADL: activity of daily living.

^d^AI: artificial intelligence.

#### Sensors

In 14 reviews, sensors were used as an assessment and diagnostic tool, as an intervention, as support for daily living, and as a monitoring solution. Sensors were the most used technology in the included reviews and were often used in combination with other technologies (Figure 2).

Thirteen groups of sensors could be identified for use in rehabilitation. The groups can be divided into 4 subgroups depending on their purpose: motion-capturing sensors (motor activity [51], posture [53], falls [140], and gait [141]); vital parameter sensors (heart rate [142], pulse, respiratory rate [143], blood oxygen [144], glucose [145], and skin and body temperature [146]); activity-tracking sensors (steps [147] and physical activity [148]); and sensors intended to capture behavior (sleep [149] and social behaviors [150]).

Sensors are embedded in wearables that are worn as normal clothes or in footwear (eg, insole pressure sensors [151]); cameras; accessories (eg, smartwatches [152], bracelets [148], rings, chest belts, and glasses [131]); or smartphones, or they can be directly attached to the skin (eg, electrodes [61]).

Inertial measurement units (IMUs) are the combination of inertial sensors, namely, the combination of one or more accelerometers, one or more gyroscopes, and, potentially, one or more magnetometers, to measure the force, angular rate, and orientation of the body [53]. These IMUs are designed to capture physical movement and posture in a markerless fashion, with the intention of detecting dysfunctions, motor impairments, activity limitations, and unhealthy conditions [47,52-54,153].

Given the existence of IoT, sensors can collect data and transfer them to other devices to visualize the output and thus provide biofeedback to the client. Biofeedback systems consist of an input sensor, a data-processing system, and an output device that displays the feedback [50]. The output can be provided through the visualization displayed on a screen, auditory feedback via voice outputs, or vibrotactile feedback of the input sensor. For example, robotic gloves can be used as sensor gloves equipped with flex sensors and vibrotactile motors that provide vibrotactile feedback through the motors at the fingertips. Sometimes, biofeedback systems are embedded in games for rehabilitation purposes in which the client must playfully perform movement tasks that are displayed on a screen. Biofeedback can improve outcomes by engaging clients and has the potential to support clients in targeted exercises during home-based rehabilitation [50].

It is useful to apply vital parameter sensors to monitor the health status of clients during rehabilitation. Kwon et al [144] developed an app for clients with COPD and monitored their heart rates and blood oxygen saturation via sensors embedded in smartphones. An alarm function alerts the client regarding any critical health status during physical activity, such as when the values (S_P_O_2_ and heart rate) fall below certain thresholds. Thus, the client has the opportunity for self-monitoring, that is, to receive feedback on the correlation between physical activity and body reactions to adjust such behavior as needed. Health care professionals can also estimate these data using a dashboard where the data are collected. After 6 weeks, the application led to a significant reduction in symptoms associated with COPD compared with the control group. The intervention group’s self-assessment of the impact of the disease also improved significantly.

Overall, stakeholders emphasized the importance of sensors in DR.

#### Robotics and Brain-Computer Interfaces

In total, 9 reviews investigated the use and effects of robots in rehabilitation. Furthermore, 9 articles that explained the use of brain-computer interfaces (BCIs) in rehabilitation were included.

Robotic gloves [154] are categorized as rehabilitation robots, and they are machines with sensors and actuators [40]. There are several types of robotic gloves [154]. For example, soft robotic gloves are used in upper-limb rehabilitation for clients with neurological conditions, such as stroke and spinal cord injury. These are used to assist, replace, or promote the movement of fingers, hands, and wrists and to facilitate activities of daily living (ADLs) [155]. They can also be used for motion capture to assess arm function and movement control [156]. Robotic gloves have the potential to be easily used by someone who is at home alone.

Exoskeletons are wearable robotic units controlled by computer boards [157,158]. A distinction is made between “rigid” and “soft” exoskeletons. Because of the perspective of home-based rehabilitation in this review, only soft exoskeletons were used in the identified references. Exoskeletons can be applied to the upper or lower limbs in home-based rehabilitation to assist clients and help perform passive movements in clients with neurological diseases such as stroke, spinal cord injuries, and cerebral palsy [159]. Furthermore, these devices can assist in the ADLs.

The application of noninvasive BCIs has changed over the last decade. This change allows health care professionals to offer this treatment in a home-based setting, although this approach remains under development. In the past, the application of BCI technologies in a home environment was hampered by the fact that the operation of these systems required the supervision of an expert. In a home-based setting, BCI technologies are used for people with severe motor impairments, such as those who have experienced a stroke, traumatic brain injury, spinal cord injury, or locked-in syndrome. These technologies can help such clients to enable or support communication with their environment or performing daily tasks.

BCI-based applications can be used to control daily aids such as wheelchairs, prosthetics, video games, and various computer applications [72,75]. Yang et al [75] proposed a system that helps physically disordered people to control external devices using gazing and eye blinks. It offers the opportunity to conduct routine daily tasks using brain signals directly, without any physical movement.

Zulauf-Czaja et al [76] developed a system for hand rehabilitation based on a BCI interface that uses an electroencephalographic device combined with functional electrical stimulation. The hand therapy consisted of the attempted movement of one hand to lower the power of the sensory-motor electroencephalography and thereby activate the functional electrical stimulation, which causes flexion and extension of the wrist.

Stakeholders saw high potential in the use of exoskeletons in rehabilitation if the devices are able to make their own adjustments in terms of speed and level of assistance based on data collected from the client. Furthermore, stakeholders indicated rehabilitation robots’ low accessibility to clients, as these are mostly available at research level. The stakeholders also emphasized the possibility of the combined use of robotic devices with gamification to encourage clients’ motivation during the rehabilitation process due to gaming elements or appealing environments.

#### Gamification

In 19 reviews, games were used either specifically for rehabilitation (serious games: n=4, 21%) or commercial use (exergames: n=15, 79%).

Gamification was mostly used as an intervention in neurological rehabilitation to improve physical function, balance, gait [84,90], and motor function [89,92]. One review applied gaming elements in rehabilitation to improve cognitive function in patients with neurological disorders (stroke, MS, and cognitive impairment) [91]. Mura et al [91] stated that no conclusion can be drawn regarding the effectiveness of exergames in improving the cognitive function in persons with neurological disabilities. The small sample sizes of the selected studies, dissimilarity in outcomes and assessments used to measure the cognitive function, and heterogeneity of populations included in the analysis restricted the explanatory power of the review.

In a number of reviews [54,78-80] in which serious games were described, hardware such as robotic devices and gloves and leap motion sensors were used as supplements to the game.

Publications deployed exergames using commercial hardware, such as the PlayStation, Nintendo Wii, Xbox Kinect, and associated elements such as the Wii balance board and games for these consoles [59,83,84,88-90,92,160]. Biomarkers such as leap motion sensors and Microsoft Kinect are used in motion capturing and creating physical images on the screen.

Overall, a low strength of evidence has been shown until now regarding exergames and serious games in improving physical functioning. This is due to a lack of long-term randomized controlled trials (RCTs) with homogeneous population and large sample sizes and specific outcome measures [80,82,84,88,89,91,92]. Stakeholders also stated that the long-term effects of gamification are yet to be proven.

#### VR and AR Technologies

For the recent past and as this review shows, more VR technologies (5/7, 71%) have been the focus of research in relation to rehabilitation compared with AR technologies (2/7, 29%).

In home-based rehabilitation, VR and AR are used to improve physical functioning [93], balance [161] and anxiety [162] in older individuals and people with neurological diseases. It was further revealed that VR is used by people with vestibular dysfunction to improve balance and postural control [96].

VR technologies were mostly used in combination with games that had not been developed for rehabilitation purposes, with the exception of 6 studies [161-166]. In the included studies, AR was used in combination with other devices, such as a robotic glove [167] or motion-capturing devices [168], to display an avatar on a screen, allowing clients to see their own movements. All software applications used for AR were developed for research purposes.

In 1 study [167], AR was used as a mirror therapy in combination with a robotic glove to improve the motor function of paretic limbs in clients after stroke. Participants saw a mirror image of themselves on a screen, with the paralyzed arm being mimicked by the virtual arm. They were instructed to perform tasks by first moving the uninjured arm and then the paralyzed arm—the animation of the virtual arm—was triggered by signals from markers on the glove.

In addition to VR and AR, stakeholders added “Mixed reality” and “Digital twins” as a further trend in rehabilitation. Mixed reality is the merging of real and virtual worlds to produce new environments and visualizations in which physical and digital objects coexist and interact in real time. It includes VR and AR and thus represents the entire spectrum between the physical and digital worlds [38]. A digital twin is a digital representation of tools, people, processes, and systems. In rehabilitation, digital twins are used to create digital representations of health-related data, such as data regarding hospital environments, laboratory results, and human physiology, through computer models [169]. No application of digital twins in home-based rehabilitation has been reported. However, digital twins are not yet applicable in home-based settings.

Currently, there is a lack of strong evidence supporting the use of VR and AR for rehabilitation [49]. Stakeholders summarized that VR emerged in the market for rehabilitation 10 years ago and that the use of VR had been rather limited in the past because of its low usability. They further indicated that AR had greater potential than VR.

#### Digital and Mobile Apps

In this study, we identified 3 subcategories within the 26 reviews regarding digital and mobile apps: apps (19/43, 44%), web-based programs (17/43, 40%), and videoconference systems (7/43, 16%). The large number of published papers in this area illustrates their broad use in rehabilitation.

Apps, web-based programs, and videoconference systems can be used for remote and home-based rehabilitation as adjuncts to face-to-face therapy or to replace some parts of it. It is noticeable that the use of these technologies is not limited to certain target groups, rather the client must fulfill some preconditions to use these technologies, such as access to the internet, mobile devices, or computers, as well as possessing necessary skills.

Apps and web-based programs are used as assessments, specifically providing questionnaires via apps, or delivering guidance regarding assessments, or measuring movement through special sensors embedded in a smartphone [52,106,170-172]. These technologies can promote physical activity; improve physical functioning, mobility, and language and speech skills; and provide secondary prevention through exercises and (real-time) feedback, information, (self-) monitoring, and reminder functions [105,111,114].

Moreover, these technologies are used to improve cognitive functions [60,173] and provide psychological support [102]. Two articles [174,175] additionally described the function of web-based programs to improve balance, strength, mobility, and postural control of people with MS by providing physical exercise.

Apps and web-based programs can help improve self-management and encourage a healthy lifestyle, rehabilitation, and adherence through individual goal setting, displaying rehabilitation progress, showing motivational messages, providing educational modules, and enabling symptom recording and social support [49,52,108,109,112].

Digital and mobile apps can further offer the possibility to connect with other people and become part of a social support community [176].

Stakeholders have criticized the lack of a solution that combines all the requirements of the rehabilitation process in one holistic approach. Even if the applications are available at a low price, the stakeholders emphasized that equal access to the internet and hardware should be ensured to decrease social inequality. They also expressed concerns that some clients may be excluded because they did not have the required competences and skills. Appropriate support is required for these clients. Furthermore, stakeholders have critically noted that digital and mobile apps encourage the replacement of face-to-face interventions, which they believe represents a clear disadvantage in terms of the outcomes of the rehabilitation process. In addition to all the benefits and limitations of digital and mobile apps, stakeholders identified this technology as one of the trends in the future health care sector.

#### IoT Principles

In total, 4 reviews explored the functionality of IoT principles.

In rehabilitation, the IoT is beneficial for the collection of clients’ data through various technologies. These data can be sent to health care professionals to monitor clients’ health in their normal environment. In this manner, data can be collected to provide a complete picture of clients without blind spots. This information can be used to make medical decisions and treatment plans.

IoT approaches include smart homes and ambient-assisted living (AAL). A smart home is a residence equipped with smart technologies aimed at providing tailored services to clients [177]. AAL can be defined as the use of information and communication technologies in a person’s daily living and work environment [178]. Smart homes and AAL make it possible to monitor and support residents in ADLs or create a safe environment, which can enhance quality of life and promote independent living. For example, in 1 project [179], the client’s house was equipped with intelligent sensors to monitor the client and provide reminder to perform tasks such as taking medication or resting.

Piau et al [58] raised the question of whether the principles of smart homes can be applied to detect mild cognitive impairment or dementia in older individuals. For this purpose, data were gathered through either digital biomarkers, such as passive sensors, that were installed in homes (motion, light, temperature, and activity sensors) or wearables. Data from dedicated or purposive technological solutions can be used to monitor a client’s activities. Nevertheless, the authors concluded that most technologies were far removed from everyday life experiences and were not sufficiently mature for use under nonoptimal or uncontrolled conditions.

Stakeholders emphasized the complexity of using the devices and high costs of the technologies, as well as expenses for installation, repair, and maintenance, as further barriers to implementing smart homes [177].

Referring to IoT, another approach that could benefit rehabilitation is living labs. The term “living labs” refers to the use of sensors to objectively record and evaluate people’s behavior and physical functioning without interruption over a long period [123]. Personal or ambient technologies can be used for this purpose. Personal technologies include wearables that are attached to clothing or rest on the skin, such as smart watches or bracelets. Ambient technologies are placed in a client’s home, such as cameras and pressure and motion sensors, which are almost not perceived during use. These technologies can record various vital parameters, as well as collect information about physical activity, muscle activity, falls, and sleeping behavior.

Stakeholders emphasized the benefits of linking the digital and physical worlds through IoT to enable a holistic approach to rehabilitation. In contrast, stakeholders have stated that clients often have great fears about the use of these technologies with an IoT approach owing to lack of safety.

#### AI and Machine Learning

In total, 4 reviews describing the principles of AI were included. In addition, 13 articles were selected to explore its use in rehabilitation with machine learning processes.

AI and machine learning processes can be used as diagnostic and prognostic tools in rehabilitation. Abdollahi et al [124] used a wearable system of sensors (IMUs) and machine learning processes to classify clients with nonspecific low back pain into subgroups according to quantitative kinematic data, for example, trunk motion– and balance-related measures. On the basis of this home assessment, a personalized rehabilitation plan was created following practical guidelines.

Similarly, several articles have developed a home-based monitoring system based on AI and machine learning for use in executing a rehabilitation plan, even in the absence of a rehabilitation professional. For example, Chae et al [127] developed a home-based rehabilitation system on a smartwatch, as well as an app and AI processes that can recognize and record the type and frequency of rehabilitation exercises conducted by the client. This can facilitate participation in home training and improve the functional scores of patients with chronic stroke. Lydakis et al [131] designed a system of wearable glasses and smart bracelets to provide interactive corrective feedback to clients with neurological diseases in home settings.

Chatbots and conversational agents are key technologies for AI and machine learning processes in rehabilitation. They could be based on AI and machine learning processes that simulate and process human conversations. They enable communication via text or audio on websites, mobile apps, or telephone [137].

Vaidyam et al [137] further identified the potential of chatbots for use in psychoeducation and for encouraging self-adherence by providing information and motivation. The RCTs performed by Fitzpatrick et al [180] and Fulmer et al [99] reported health-related outcomes. They found that interaction with conversational agents led to decreased symptoms of depression and anxiety compared with the control groups.

In their reviews, Schachner et al [139] and Vaidyam et al [137] found that the acceptance rating of conversational agents and chatbots was positive, suggesting that they would be effective and enjoyable tools for use in rehabilitation.

Virtual human technology is based on AI and machine learning processes and used in rehabilitation. Virtual humans are computer-generated cartoon-like characters that have the ability to initiate and respond to verbal and nonverbal communications. The use of virtual humans in assisted care has mainly been implemented for healthy participants [181-183], with the aim of improving their health behaviors and reducing risk factors or physical inactivity. Furthermore, they have been used to provide advice and serve as motivators to increase physical activity in older individuals [184].

From a systemic perspective, the stakeholders expressed that AI and machine learning processes are the most relevant technologies for the future. AI and machine learning processes define future opportunities in the rehabilitation sector because they enable evidence to be generated based on the data collected. This can lead to resource optimization for the client and the health care system. However, stakeholders have stated that technologies such as chatbots and conversational agents have not yet been sufficiently developed for use in rehabilitation. Most of the work is still in the pilot phase, and the effectiveness of the technology has not yet been verified [139].

### Research Question 3: Influence of COVID-19 on the Development of DR

The 3-year pandemic (with May 5, 2023, as the end) has changed many rehabilitation processes for clients and health care professionals. To present the influence of the COVID-19 pandemic on DR, 40 records were included.

To continue rehabilitation during the pandemic, remote digitally driven rehabilitation approaches, such as videoconferencing, apps, and web-based programs, were frequently used in practice. However, the crisis did not lead to the development of new DR technologies but rather to an increased use of technologies that were already on the market.

This has led to more experiences with DR among clients, health care professionals, and health care providers. Clients accepted DR remotely as a substitute for face-to-face therapy during the pandemic [110,185-188]. However, they also identified barriers to the use of technologies [186,188,189]. To increase clients’ adoption, recommendations have been developed [110]. Because many health care professionals are not trained to use virtual approaches in low-income countries [190], as well as in industrialized countries [191], additional training was offered to build competences in this area. Bernocchi et al [192] further stated that not only clients but also health care professionals require adequate training to use digital approaches.

The pandemic has led to an increased awareness of the need to develop further digital health measures (eg, digital vaccination certificate, digital sick note, improvement of digital tools, and simplified access to digital processing). In many countries, efforts have been made to implement digital solutions in the health care sector or help meet the preconditions for their use. However, the COVID-19 pandemic acted as an additional driver in the implementation of these applications. This was supported by political measures such as the softening of strict guidelines, which made it possible to launch and use digital solutions even without proof of their effectiveness [193]. However, clients have expressed concerns regarding their effectiveness and safety [194].

In addition, in some countries (Austria and Germany), videoconference (therapy via videoconferencing) was legally approved, considering individual requirements, such as a specific diagnosis, the confirmation of the service provided, safe technical equipment, and a positive prognosis regarding the success of the application [195,196]. In Germany, this permission remains permanent regardless of the pandemic situation [197].

However, the rapid implementation has also led to the frequent implementation of incomplete solutions that have met the clients’ short-term needs but are not sustainable in the long term, as pointed out by stakeholders. In addition, evaluations of the measures have been less rigorous or have not been conducted, leading to the risk of perpetuating rapidly implemented solutions after the pandemic [193].

## Discussion

### Principal Findings

This scoping review revealed various digital technologies intended for use in home-based rehabilitation. Trends were identified, and the advantages and disadvantages of each technology were presented. In addition, the influences of the pandemic on the development of DR were shown.

The findings of this review reveal that sensors, robotic devices, gamification, VR and AR, and digital and mobile apps are already widely used in home-based rehabilitation. However, AI and machine learning, exoskeletons, and digital and mobile apps represent emerging trends in rehabilitation. Compared with the other identified technologies, VR, AR, and robotics cannot be used independently for home-based rehabilitation for usability and safety reasons. Thus, there is a need to develop sufficient and feasible DR practices that demonstrate clinically relevant effectiveness. Furthermore, we discovered that the COVID-19 pandemic has led to an increased use of digital technologies in a remote approach, especially apps, web-based programs, and videoconferencing, but not to the development of new technologies. Clients and health care professionals accepted this approach during the pandemic, but they also expressed concerns about it. The pandemic acted as a driver for implementing remote approaches for health care systems. However, sustainable solutions that can be applied even after the pandemic should be implemented.

One major finding was that the relevance of AI and machine learning will increase in the field of rehabilitation in the future, especially for diagnostic procedures, decision-making, and the development of client-centered care, even though they are not yet broadly applied in rehabilitation. Furthermore, it was found that AI, machine learning, and digital and mobile apps would be essential to process and optimize resources in the health care sector. This finding is consistent with that in the current literature. For example, Hamet and Trembley [198] stated that electronic medical or health records are essential tools for use in personalized medicine, early detection, and targeted prevention, with the aim of increasing their clinical value and decreasing health costs. Moreover, Róman-Belmonte et al [199], who investigated the influence of AI on musculoskeletal disorders, highlighted that AI can produce a paradigm shift in musculoskeletal health, a move from descriptive to predictive medicine.

Nevertheless, there are challenges that need to be overcome when applying AI and machine learning widely in the health care sector. Jiang et al [200] report a lack of ethical and legal supervision. No globally unified laws or regulations regarding the application of AI in medicine are currently in place to standardize the behavior of practitioners [200]. In addition to ethical and legal issues, one major challenge is the clear need for a standardized, comparative evaluation of the effects of AI on health indicators and measures of changes in psychological and physical status, side effects, and outcomes [198]. The findings regarding acceptance were incoherent. Health care professionals support the application of AI in rehabilitation and desire training for its application. However, people who are less well informed about AI fear being replaced by this technology, often because of a lack of knowledge about AI [200]. Therefore, it is important to outline the benefits and barriers of using AI for rehabilitation within society.

One further result was that technologies such as gamification, VR and AR, and digital and mobile apps that are already used in home-based rehabilitation have the potential to improve client adherence and motivation. Similarly, approaches based on IoT can increase client participation in the rehabilitation process because they allow self-monitoring; thus, client self-management can be increased. This represents an important factor for successful therapy of chronic diseases to improve quality of life and reduce the use of health care resources [201].

This study revealed that most technologies could be used in home-based rehabilitation without the presence of health care professionals. The emerging developments in the design and possibility of asynchronous or synchronous monitoring via apps, web-based programs, or videoconferencing make the remote rehabilitation approach possible. In other studies [54,56,202], the results showed that the independent use of VR and AR technologies and robotics in a home setting is limited owing to low usability and safety concerns. Clients with disabilities may require assistance in attaching or using the device or face the risk of harming themselves when excessive force is transferred to the body. However, to provide independent, usable, and safe rehabilitation at home, trained caregivers supporting the clients [76,203] or the construction of the rehabilitation device have the ability to overcome this challenge. For example, attachment mechanisms could be designed to enable the client to don and doff the device [159]. Furthermore, Kim et al [204] used emergency stop buttons and added safety features limiting ROM and joint velocity and stopping the robot in case of excessive force and torque interaction.

In order to offer client-friendly rehabilitation in the home setting, sufficient space for the equipment must also be ensured. Furthermore, the technical equipment needs to meet requirements such as access to the internet or a digital and mobile device, and the costs of purchasing, repairing, and maintaining the technology need to be low [62] because such costs are not always covered by health insurance funds.

In addition to the safety and usability factors, the use of DR should be based on an individual rehabilitation goal, considering the motivation of clients and health care professionals to use technologies and the possibility of receiving support when using such technologies [205]. Furthermore, individual physical conditions should be considered, which may affect the use of the selected therapy (grade of dementia, disability of vision or hearing, and degree of impairment). It must be noted that Cottrell and Russell [205] limited their recommendations to videoconferencing in physiotherapy practice. Given that the technology must be customized to the client’s body, these recommendations can also be considered when using other technologies.

Another major finding is that the effectiveness of many technologies has not yet been confirmed scientifically. Several studies have described projects in which the technology is currently under development or tested in laboratory settings. However, it can also be seen that 221 RCTs referring to DR are already registered via ClinicalTrials.gov for 2021 and 2022 (September 15, 2022). Because many technologies have only been used under controlled conditions, their application in daily life cannot be assessed.

In addition, we found that feasible approaches to the implementation and integration of digital technologies for the rehabilitation process are lacking. Accordingly, it is essential to consider not only the effectiveness of the intervention in terms of dedicated outcomes, the design of the intervention itself, and the characteristics of individuals but also the setting in which the approach will be applied, the process of implementation, and factors influenced by policies and government regulations [206].

Therefore, studies are required that present an effective and valid concept that can enable clients and health care professionals to apply digital technologies for (home-based) rehabilitation. This includes the presentation of plans for the modus of DR (blended therapy or replacing face-to-face therapy), the reimbursement of health care professionals, and cost coverage for the technologies needed by clients and rehabilitation units. Other issues must be addressed for a feasible implementation, such as privacy compliance, data protection, insurance coverage, and the assumption of responsibility and liability if harm occurs during remote or unsupervised rehabilitation.

The social distancing regulations enacted during the COVID-19 pandemic caused many clients and health care professionals to use DR, especially videoconferencing, apps, and web-based programs. Clients and professionals described their relatively high level of DR positivity along with improvements over the course of the COVID-19 lockdown. However, the question of whether this therapy model will continue to be accepted without exceptional circumstances remains.

The literature shows contrary results regarding the acceptance of digital interventions by clients and health care professionals [13,207]. However, it is noteworthy that many terminologies exist for *Digital Rehabilitation* that are associated with various technologies. It is likely that the acceptance rates of different technologies vary, which does not allow for a general statement about their acceptance among clients and health care professionals.

Through the COVID-19 pandemic, more training opportunities for health care professionals were offered to allow them to gain competence in the field of DR. To be adequately prepared for working life and systematize the acquisition of competencies, health care professionals should already have acquired the necessary skills in higher education. Because DR technologies have already been implemented in rehabilitation, it is crucial that health care professionals are sensitized to the possibility of integrating digital options, acquire useful competencies, and have the ability to recognize which clients will benefit from this approach and then provide adequate assistance to them.

Although the pandemic was, in many ways, a driver of the use of DR, it also led to the rapid introduction of solutions that were not based on an efficient concept and left many questions unanswered with regard to realistic implementation. Therefore, careful planning with a phased, linear implementation approach is crucial for establishing sustainable DR practices that will last beyond the pandemic and have the potential to meet the challenges of rehabilitation in the future [208].

The results of this study paint a very broad picture of existing and emerging technologies in rehabilitation because a large number of records were included in the analyses, which were not limited to a specific target group or rehabilitation outcomes. The results derived from the scientific databases were complemented by a search of the gray literature. Through a meeting with key stakeholders, insights were gained beyond those reported in the literature. In addition to health care professionals, politicians, experts in the field of digitalization in rehabilitation, and clients with experience or a specific interest in DR were included.

### Limitations

Bibliometric and co-occurrence analyses were performed with a minimum of 10 occurrences per keyword. This may have influenced the completeness of the representation of technologies if newer technologies occurred fewer than 10 times throughout the data set. However, the stakeholder meeting served to close this gap. Moreover, we did not weigh the strength of the evidence from all papers or appraise the efficacy of the approaches, which would have been beyond the scope of this review. In addition, new technologies may have been developed in the meantime and were not presented here because of the rapid development of the market.

In the search for relevant literature, we used the word “rehabilitation” and not other terms commonly used in the context, such as “therapy,” “training,” “intervention,” or “treatment,” because we wanted to stick to the concept of rehabilitation, which implies a multimodal, collaborative, and patient-centered process rather than the stand-alone interventions suggested by those terms [209]. However, it resulted in few articles being included related to mental and psychosocial disorders, despite the Cochrane definition of rehabilitation states that rehabilitation also includes people with mental problems [209]. Thus, rehabilitation seems to be still more closely associated with the improvement or optimization of physical functions.

### Conclusions

These findings reflect the growing interest in the use of digital technologies in rehabilitation. Multiple tools are already available and implemented for home-based rehabilitation; however, there are some limitations to their use, such as low usability, safety concerns, ethical challenges, and a lack of efficacy and legal frameworks. DR implementation should be based on the clients’ goals and motivation. AI and machine learning could be of particular interest in redesigning rehabilitation to address future challenges in the rehabilitation sector.

On the one hand, the pandemic acted as a driver for the application and acceptance of existing digital solutions in rehabilitation. On the other hand, digital solutions that only met the requirements of the clients during the pandemic were implemented. The results of this research reflect the need for feasible and effective approaches to implement DR sufficiently to meet clients’ needs and adhere to framework conditions to be sustained apart from exceptional situations.

## Appendix

Multimedia Appendix 1 PRISMA-ScR (Preferred Reporting Items for Systematic Reviews and Meta-Analyses extension for Scoping Reviews) checklist.

Multimedia Appendix 2 Search strategies.

Multimedia Appendix 3 Definitions.

## Abbreviations

AAL: ambient-assisted living
ADL: activity of daily living
AI: artificial intelligence
AR: augmented reality
BCI: brain-computer interface
COPD: chronic obstructive pulmonary disease
DIRENE: Competences for the new era of user-driven Digital Rehabilitation
DR: digital rehabilitation
IMU: inertial measurement unit
IoT: Internet of Things
MS: multiple sclerosis
PRISMA-ScR: Preferred Reporting Items for Systematic Reviews and Meta-Analyses extension for Scoping Reviews
RCT: randomized controlled trial
RIS: Research Information Systems
VR: virtual reality

## Data Availability

The data sets generated and analyzed during this study are available from the corresponding author upon reasonable request.
