# Supplementary material for: Technologies in Home-Based Digital Rehabilitation: Scoping Review
Source: JMIR Rehabil Assist Technol. 2023 Jul 27;10:e43615. doi: 10.2196/43615 (PMC10415951; doi:10.2196/43615)
Supplement: Multimedia Appendix 2 [file rehab_v10i1e43615_app2.docx]

# Multimedia Appendix 2 Search strategies

#### PubMed

rehabilitation [tiab] OR "Physical and Rehabilitation Medicine"[Mesh]

AND

care at home [tiab] OR home* [tiab] OR homebased [tiab] OR home-based [tiab] OR house* [tiab] OR distance[tiab] OR distant [tiab] OR domicile [tiab]

AND

advanced medical technology [tiab] OR artificial intelligence [tiab] OR application [tiab] OR augmented reality [tiab] OR communication [tiab] OR connected health [tiab] OR computer-based [tiab] OR computer-assisted [tiab] OR computer-aided [tiab] OR computerbased [tiab] OR computerassisted [tiab] OR computeraided [tiab] OR **digital[tiab] OR ehealth[tiab] OR e-health[tiab] OR electronic health[tiab] OR e-diagnosis[tiab] OR e-intervention[tiab] OR „e-therapy"[tiab] games[tiab] OR high-tech medical technology[tiab] OR human-computer interface[tiab] OR hybrid[tiab] OR "information and communication technology"[tiab] OR "Information Technology"[Mesh] OR internet-assisted[tiab] OR internetassisted[tiab] OR internet-based[tiab] OR internetbased[tiab] OR internetaided[tiab] OR internet-aided[tiab] OR medical technology [tiab] OR mhealth[tiab] OR m-health[tiab] OR mobile health[tiab] OR "Mobile Applications"[Mesh] OR mobile[tiab] OR online[tiab] OR remote[tiab] OR robot*[tiab] OR "Robotics"[Mesh] OR telerobot*[tiab] OR tele-robot*[tiab] OR smartphone[tiab] OR technolog*[tiab] OR tele-technolog*[tiab] OR tele-homecare[tiab] OR tele-diagnosis[tiab] OR tele-care[tiab] OR "Telerehabilitation“[Mesh] OR tele-rehabilitation[tiab] OR "Telemedicine"[Mesh] OR tele health[tiab] OR tele medicine[tiab] OR telecare[tiab] OR teleconsultation[tiab] OR telediagnosis[tiab] OR telehealth[tiab] OR telehomecare[tiab] OR telemedicine[tiab] OR teletechnology[tiab] OR "Telemetry"[Mesh] OR telemetry[tiab] OR telemonitor*[tiab] OR teletherapy[tiab] OR televideo[tiab] OR "Therapy, Computer-Assisted"[Mesh] OR "User-Computer Interface"[Mesh] OR video-consultation[tiab] OR videoconferencing[tiab] OR "Videoconferencing"[Mesh] OR "Video Games"[Mesh] OR video game [tiab] OR videogame[tiab] OR video-teleconference[tiab] OR videoteleconference[tiab] OR virtual*[tiab] OR wearable[tiab] OR web-based[tiab] OR web-assisted[tiab] OR "web-supported“[tiab] OR webbased[tiab] OR webassisted[tiab] OR websupported[tiab]**

**First search: Filters: English, published from 2015/01/01/– 2021**

Search was conducted on 23th July 2021

Second search: **Filters: English, published from 2021/07/24/– 2022**

Search was conducted on 24^th^ January 2022

#### Embase via Ovid

*rehabilitation/ or *rehabilitation care/ or (rehabilitation).ab,kw,ti.

**AND**

(care at home or home$ or homebased or home-based or house$ or distance or distant or domicile).ab,kw,ti.

AND

**(Advanced medical technology or application or artificial intelligence or augmented reality or communication or connected health or computer-based or computer-assisted or computer-aided or computerbased or computerassisted or computeraided or connected health or digital or ehealth or e-health or ehealth or electronic health or ehealth technology or e-diagnosis or e-intervention or e-therapy games or high-tech medical technology or human-computer interface or hybrid or internet-assisted or internetassisted or internet-based or internetbased or internetaided or internet-aided or medical technology or mhealth or m-health or mobile health or mobile or online or remote or robot$ or telerobot$ or tele-robot$ or smartphone or technolog$ or tele-technolog$ or tele-homecare or tele-diagnosis or tele-care or telerehabilitation or tele-rehabilitation or tele health or tele medicine or telecare or teleconsultation or telediagnosis or telehealth or telehomecare or telemedicine or teletechnology or telemetry or telemonitor$ or teletherapy or televideo or video-consultation or videoconferencing or video game or video-teleconference or videoteleconference or virtual$ or wearable or web-based or web-assisted or web-supported or webbased or webassisted or websupported).ab,kw,ti. or *information system/ or *information technology/ or *robotics/ or *telecommunication/ or *telehealth/ or *teleconsultation/ or *telemedicine/ or telemetry/ or *remote sensing/ or *telephone telemetry/ or *computer assisted therapy/ or *videoconferencing/ or *video game/th [Therapy]**

First search: Filters: limit 16 to yr = "2015 - 2021"

Search was conducted on 28^th^ July 2021

Second search: Filters: limit 16 to yr = "2021 - 2022"/

Search was conducted on 24^th^ January 2022

#### PsycInfo

(Rehabilitation) TI OR AB OR KW OR DE "Cognitive Rehabilitation" OR DE "Neuropsychological Rehabilitation" OR DE "Neurorehabilitation" OR DE "Occupational Therapy" OR DE "Physical Therapy" OR DE "Psychosocial Rehabilitation"

AND

(care at home OR home* OR homebased OR home-based OR house* OR distance OR distant OR domicile) TI OR AB OR KW

AND

(„advanced medical technology“ OR artificial intelligence OR application OR augmented reality OR communication OR connected health OR computer-based OR computer-assisted OR computer-aided OR computerbased OR computerassisted OR computeraided OR **digital OR ehealth OR e-health OR „electronic health“ OR e-diagnosis OR e-intervention OR „e-therapy" games OR high-tech medical technology OR human-computer interface OR hybrid OR "information and communication technology" OR "Information Technology" OR internet-assisted OR internetassisted OR internet-based OR internetbased OR internetaided OR internet-aided OR medical technology OR mhealth OR m-health OR mobile health OR "Mobile Applications" OR mobile OR online OR remote OR robot* OR "Robotics" OR telerobot* OR tele-robot* OR smartphone OR technolog* OR tele-technolog* OR tele-homecare OR tele-diagnosis OR tele-care OR "Telerehabilitation“ OR tele-rehabilitation OR "Telemedicine" OR tele health OR tele medicine OR telecare OR teleconsultation OR telediagnosis OR telehealth OR telehomecare OR telemedicine OR teletechnology OR telemetry OR telemonitor* OR teletherapy OR televideo OR "User-Computer Interface" OR video-consultation OR videoconferencing OR "Videoconferencing" OR "Video Games" OR video game OR videogame OR video-teleconference OR videoteleconference OR virtual* OR wearable OR web-based OR web-assisted OR web-supported OR webbased OR webassisted OR websupported) TI OR AB OR KW OR DE "Telerehabilitation" OR DE "Telemedicine" OR DE "Computer Assisted Therapy" OR DE "Electronic Health Services" OR DE "Online Therapy" OR DE "Teleconferencing" OR DE "Teleconsultation" OR DE "Video-Based Interventions" OR DE "Videoconferencing" OR DE "Virtual Environment" OR telemetry OR DE "Telemetry" OR DE "Robotics" OR MM "Information and Communication Technology"**

**Search was conducted on 28^th^ July 2021** and 24^th^ January 2022

#### Cumulative Index to Nursing and Allied Health Literature (CINAHL)

TI or AB (rehabilitation) OR (MH "Rehabilitation")

AND

TI or AB (care at home OR home* OR homebased OR home-based OR house* OR distance OR distant OR domicile)

AND

**TI or AB(„advanced medical technology“ OR artificial intelligence OR application OR augmented reality OR communication OR connected health OR computer-based OR computer-assisted OR computer-aided OR computerbased OR computerassisted OR computeraided OR digital OR ehealth OR e-health OR „electronic health“ OR e-diagnosis OR e-intervention OR „e-therapy" games OR high-tech medical technology OR human-computer interface OR hybrid OR "information and communication technology" OR "Information Technology" OR internet-assisted OR internetassisted OR internet-based OR internetbased OR internetaided OR internet-aided OR medical technology OR mhealth OR m-health OR mobile health OR "Mobile Applications" OR mobile OR online OR remote OR robot* OR "Robotics" OR telerobot* OR tele-robot* OR smartphone OR technolog* OR tele-technolog* OR tele-homecare OR tele-diagnosis OR tele-care OR "Telerehabilitation“ OR tele-rehabilitation OR "Telemedicine" OR tele health OR tele medicine OR telecare OR teleconsultation OR telediagnosis OR telehealth OR telehomecare OR telemedicine OR teletechnology OR telemetry OR telemonitor* OR teletherapy OR televideo OR "User-Computer Interface" OR video-consultation OR videoconferencing OR "Videoconferencing" OR "Video Games" OR video game OR videogame OR video-teleconference OR videoteleconference OR virtual* OR wearable OR web-based OR web-assisted OR web-supported OR webbased OR webassisted OR websupported) OR (MH "Telerehabilitation") OR (MH "Telehealth") OR (MH "Telemedicine") OR (MH "Therapy, Computer Assisted") OR (MH "Teleconferencing") OR (MH "Videoconferencing") OR (MH "Remote Consultation") OR (MH "Internet-Based Intervention") OR (MH "Augmented Reality") OR (MH "Virtual Reality") OR (MH "Telemetry") OR (MH "Robotics")****OR (MH "Exoskeleton Devices")**

Search was conducted on 29^th^ July and 24^th^ January 2022

#### Cochrane Central Register of Controlled Trials (CCRCT)

(rehabilitation):ti,ab,kw OR MeSH descriptor: [Rehabilitation] 1 tree(s) exploded

AND

(care at home or home* or homebased or home-based or house* or distance or distant or domicile):ti,ab,kw

AND

**("advanced medical technology" OR artificial intelligence OR application OR augmented reality OR communication OR connected health OR computer-based OR computer-assisted OR computer-aided OR computerbased OR computerassisted OR computeraided OR digital OR ehealth OR e-health OR "electronic health" OR e-diagnosis OR e-intervention OR "e-therapy" games OR high-tech medical technology OR human-computer interface OR hybrid OR "information and communication technology" OR "Information Technology" OR internet-assisted OR internetassisted OR internet-based OR internetbased OR internetaided OR internet-aided OR medical technology OR mhealth OR m-health OR mobile health OR "Mobile Applications" OR mobile OR online OR remote OR robot* OR "Robotics" OR telerobot* OR tele-robot* OR smartphone OR technolog* OR tele-technolog* OR tele-homecare OR tele-diagnosis OR tele-care OR "Telerehabilitation" OR tele-rehabilitation OR "Telemedicine" OR tele health OR tele medicine OR telecare OR teleconsultation OR telediagnosis OR telehealth OR telehomecare OR telemedicine OR teletechnology OR telemetry OR telemonitor* OR teletherapy OR televideo OR "User-Computer Interface" OR video-consultation OR videoconferencing OR "Videoconferencing" OR "Video Games" OR video game OR videogame OR video-teleconference OR videoteleconference OR virtual* OR wearable OR web-based OR web-assisted OR web-supported OR webbased OR webassisted OR websupported):ti,ab,kw OR MeSH descriptor: [Information Technology] explode all trees OR MeSH descriptor: [Information Systems] explode all trees OR MeSH descriptor: [Robotics] explode all trees OR MeSH descriptor: [Telecommunications] explode all trees OR MeSH descriptor: [Telemedicine] 2 tree(s) exploded OR MeSH descriptor: [Remote Consultation] explode all trees OR MeSH descriptor: [Therapy, Computer-Assisted] 1 tree(s) exploded OR MeSH descriptor: [Videoconferencing] explode all trees OR MeSH descriptor: [Video Games] explode all trees OR MeSH descriptor: [Telemetry] explode all trees**

First search: Filters with Publication Year from 2015 to 2021, with Cochrane Library publication date from Jan 2015 to Jul 2021, in Trials

Search was conducted on 30^th^ July 2021

Second search: Filters with Publication Year from 2021 to 2022, with Cochrane Library publication date from August 2021 to January 2022, in Trials

Search was conducted on 24^th^ January 2022
