# Supplementary material for: Technologies in Home-Based Digital Rehabilitation: Scoping Review
Source: JMIR Rehabil Assist Technol. 2023 Jul 27;10:e43615. doi: 10.2196/43615 (PMC10415951; doi:10.2196/43615)
Supplement: Multimedia Appendix 3 [file rehab_v10i1e43615_app3.docx]

## Multimedia Appendix 3

### Definitions

The consortium of the DIRENE project of the Erasmus+ program has developed the present definitions of technologies used in Digital Rehabilitation. They reflect the understanding of the consortium and were created through a Delphi process adapted based on indicated literature until consensus was reached.

| **Technology** | **Definition** |
| --- | --- |
|  |  |
| Artificial intelligence | Artificial intelligence (AI) refers to systems that display intelligent behavior by analyzing their environment and taking actions – with some degree of autonomy – to achieve specific goals. AI is a human-designed software that operates in the digital and possible physical dimension by collecting (e.g., via sensors) and evaluating data. Machine learning algorithms can be used in AI solutions, which focus on the use of data to imitate the way that humans learn, gradually improving its accuracy [29-31]. |
| Augmented reality | **Augmented reality (AR) is a user interface that combines the real world with three-dimensional and interactive elements in real time. Suitable systems are designed to display AR as if it were part of the real world. An enhanced version of the real physical world is achieved through the use of digital visual elements, sound, or other sensory stimuli delivered via technology [32].** |
| Brain-computer interface | **A brain-computer interface (BCI) is a connection between a brain and a computer or an external device that enables signals from the brain to direct various external activities without the participation of the peripheral nerve and muscles [33].** |
| Chatbots | A chatbot is a software or computer program that simulates and processes human conversation. Chatbots can be used as an user interface allowing humans to interact with digital devices. Chatbots enable communication via text or audio on websites, mobile applications, or telephone [34]. |
| Digital Rehabilitation | **Digital Rehabilitation (DR) is the use of digital technologies (by rehabilitation professionals or self-driven clients) as part of the rehabilitation process in its different phases (diagnostic/assessment, goal setting, intervention, monitoring, evaluation). Digital Rehabilitation aims to optimize functioning and reduce disability of individuals in interaction with their environment. Therefore, Digital Rehabilitation has a strong link with the empowerment of individuals and communities. This can include, but is not limited to, the use of tele- and remote rehabilitation applications and services, automatic services, robot-assisted technologies, wearable physical activity trackers, emails, video, speech, and text messaging solutions [1].** |
| Exergames | Exergames are any digital games that request physical movements and reactions [35]. |
| Gamification | Gamification is an umbrella term and is the application of game-typical elements to non-game contexts, such as rehabilitation or the work environment, to improve adherence, engagement or productivity levels [36]. |
| Internet of Things (IoT)  Mixed reality | Internet of Things (IoT) is a generic term for a network of connections between physical objects (or group of objects) that are embedded with sensors, processing ability, software, and other technologies that connect and exchange data with other devices and systems over the Internet or other communication networks [37].  **Mixed reality (MR) is the merging of real and virtual worlds to produce new environments and visualizations, where physical and digital objects co-exist and interact in real time. It includes virtual and augmented reality and thus represents the entire spectrum between the physical and digital worlds [38].** |
|  |  |
| Mobile Applications | **Mobile Applications (Apps) are software programs that are developed for and used via mobile operating systems or mobile devices such as smartphones, tablets or smartwatches. Mobile Applications serve to provide users with similar services to those accessed on PCs or laptop [39].** |
| Rehabilitation robot | A rehabilitation robots or robotic devices used in rehabilitation are machines comprising sensors and actuators that can be programmed to replace, assist, or promote human courses of action [40]. |
|  |  |
| Robotics | Robotics is the concept of transferring human-human interaction or human-physical interaction to the principles of information technology. Robotics involves design, construction, operation, and use of robots with the goal to design machines supporting humans in challenges of the real-world [41]. |
| Sensors | Sensors are devices capable of sensing and/or responding to a physical stimulus (e.g., heat, light, sound, pressure, magnetism, or a specific motion) and transmitting the resulting pulse (e.g., to measure or actuate a control). The sensor usually consists of a transducer element, which responds directly to the measured parameter and produces a usable signal output, and its related electronics. In Digital Rehabilitation, sensors are used to assess, capture, monitor and to track client’s health status and behavior [42]. |
| Serious game | A serious game is a goal-oriented application to adopt specific content or skills in an implicit way. The intention is to combine serious aspects with the playful aspects of digital games in a coherent and simultaneous way. A serious game has a challenging goal, involves some concept of scoring points, and imparts the user a skill, knowledge, or attitude that can be applied in the real world. Serious games can be applied in almost any field: healthcare, education, politics or culture [43,44]. |
| Virtual reality | Virtual reality (VR) is an interface involving real-time stimulation and interactions of an embedded subject through multiple sensorial channels, based on a synthetic environment in which the subject feels their presence (e.g. a helmet with a screen inside, or gloves fitted with sensors) [45]. |
| Wearables | Wearables are technical devices that are worn as normal clothes or accessories, and provide data about the user via sensors. These sensors can measure quantitative data of human movement, biometric and behavioral data [46]. |
